# Cellular Signal Mechanisms of Reward-Related Plasticity in the Hippocampus

**DOI:** 10.1155/2012/945373

**Published:** 2012-11-13

**Authors:** Masako Isokawa

**Affiliations:** Department of Biomedicine, College of Biomedical Sciences and Health Professions, The University of Texas at Brownsville, 80 Fort Brown, Brownsville, TX 78520, USA

## Abstract

The hippocampus has the extraordinary capacity to process and store information. Consequently, there is an intense interest in the mechanisms that underline learning and memory. Synaptic plasticity has been hypothesized to be the neuronal substrate for learning. Ca^2+^ and Ca^2+^-activated kinases control cellular processes of most forms of hippocampal synapse plasticity. In this paper, I aim to integrate our current understanding of Ca^2+^-mediated synaptic plasticity and metaplasticity in motivational and reward-related learning in the hippocampus. I will introduce two representative neuromodulators that are widely studied in reward-related learning (e.g., ghrelin and endocannabinoids) and show how they might contribute to hippocampal neuron activities and Ca^2+^-mediated signaling processes in synaptic plasticity. Additionally, I will discuss functional significance of these two systems and their signaling pathways for its relevance to maladaptive reward learning leading to addiction.

## 1. Introduction

### 1.1. Transmitters and Modulators Involved in Reward-Related Learning

**Figure 1:**
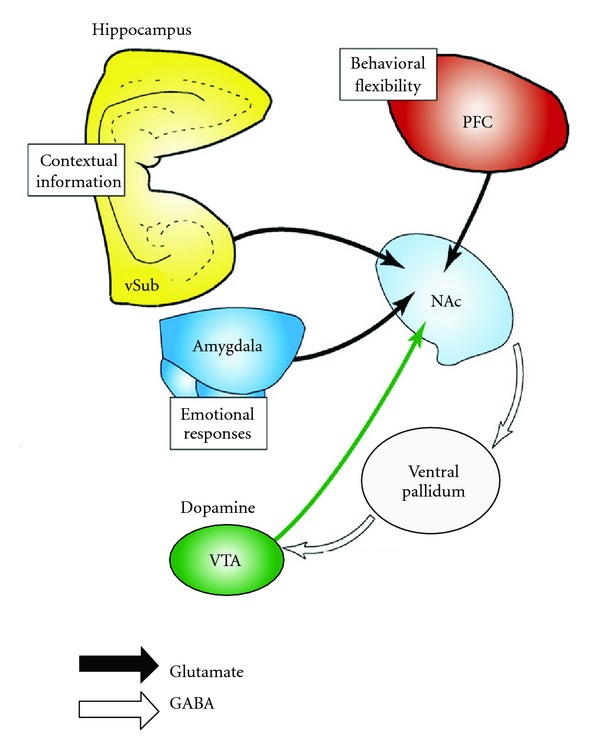
Hippocampal glutamatergic outputs regulate reward responses in the nucleus accumbens (modified from [1]). NAc: nucleus accumbens, PFC: prefrontal cortex, vSub: ventral subiculum, VTA: ventral tegmental area (modified from [1]).

Although the dopaminergic system is central to the study of motivational and reward-related learning, the neurobiological basis of reward learning and memory cannot be explained completely without the participation of the endogenous cannabinoid system and glutamatergic neurotransmission. The hippocampus, which sends a major output to the reward system, is a primary site of activity-dependent plasticity and neuromodulation, particularly by endocannabinoids and glutamate. Because the hippocampus lies upstream of the striatal dopaminergic reward circuit, cellular and synaptic plasticity within the hippocampus alters the transfer of information throughout the brain's reward system (Figure 1, also see [1]). 

Endocannabinoids are intimately involved in appetitive, motivational, and reward behavior. Endocannabinoids stimulate appetite in the hypothalamus initiating feeding behavior [2]. Moreover, endocannabinoids control consumption of substances of abuse including nicotine acting on the brain's reward system by interacting with dopaminergic, glutamatergic, and GABAergic neurons. Indeed, numerous studies have suggested the involvement of the endocannabinoid system in addiction [3]. In these studies, it was suggested that endocannabinoids may not participate in the primary reinforcing effects of substances of abuse, but are important for maintaining drug-seeking behavior. 

A hallmark of addiction is craving and relapse in the absence of substances of abuse in the organism. Craving and relapse are based on memories of the effects produced by substances of abuse on mental and physical conditions, which suggests indispensable roles of the hippocampus and the hippocampal endocannabinoid system in reward-related learning and addiction. 

Recent evidence suggests a metabolic hormone, ghrelin, may enhance hippocampal synaptic plasticity [4]. Ghrelin is a unique acylated 28 amino acid peptide that was first identified in rat stomach extracts. Ghrelin is released when the stomach is empty. It crosses the blood-brain barrier at the hypothalamus, stimulates orexigenic neurons, and initiates feeding behavior. In addition, ghrelin stimulates “reward centers” of the brain that have been linked to drug-seeking behavior. Human subjects injected with ghrelin remember pictures of food more clearly a day later and inhibitors of the ghrelin receptor could impair those memories [5]. However, to date, whether ghrelin affects memory in general or only memories pertaining to food remains unknown.

Although it is speculative, the majority of experimental research on animal models of learning might have involved ghrelin. Scientists and researchers typically put experimental animals on fasted conditions in order to facilitate the successful acquisition of specific tasks, except for one-trial learning that leads animals to learn how to avoid a negative consequence or life-threatening situation. Accelerated acquisition of learning under the fasted condition suggests potential importance of ghrelin in the motivational and reward learning in the hippocampus. 

### 1.2. Addiction Is a Maladaptive form of Reward Learning

Sometime in the history of mankind, individuals and cultures began to incorporate psychoactive drugs and alcohol use in daily life. These behaviors likely evolved from incidental exposure to compounds in wild plants while foraging. Aborigines in Australia, Thailand, and Africa made use of indigenous nicotine-containing plants and the coca plant and chewed betel nut. Fermenting alcohol has been cultivated by human societies for over 6000 years [6]. 

Clearly, whether encountered by foraging or purposefully cultivated, psychoactive drugs are by definition reinforcing. Those behaviors will be repeated in order to obtain these substances. Drugs serving as reenforcers are not a uniquely human phenomenon. Many species such as rats, mice, and nonhuman primates will directly self-administer most drugs that are used or abused by humans, such as alcohol, heroin, opiates, cannabinoids, nicotine, cocaine, amphetamine, and caffeine. Animals will perform an operant response—for example, pressing a lever—in order to obtain an intravenous infusion of these compounds. It is remarkable that 5-day-old rat pups learn to prefer odors that have been associated with morphine [7] and crayfish show positive place conditioning to psychostimulants [8]. 

It must be noted that in all these examples, learning has occurred. That is, the organism shows an adaptation in behavior that presumably reflects some level of reward value of the drug, or more precisely, the value of the state that it induces. This suggests not only that there are common chemical and molecular substrates that rewarding drugs access across species and phyla, but it also suggests that a critical feature of drug-organism interactions is plasticity [9].

The brain uses basic cellular mechanisms involving dopamine, glutamate, and their intracellular signaling cascades in order to optimize responses that ultimately enhance survival; it is clearly highly adaptive to learn where or under what circumstances food is found or danger encountered and to alter behavioral actions accordingly. Many drugs of abuse exert their primary effects precisely on these pathways and are apparently able to induce very long-term, perhaps even permanent, alterations in motivational learning networks, thus leading to maladaptive behaviors [10].

### 1.3. Cooperative Activities of Glutamate and Dopamine Can Fundamentally Alter the Behavior of the Neuron and of the Network

The hippocampus projects extensively to the striatum, using glutamate as the primary neurotransmitter. The hippocampus and the striatum also express high levels of glutamate receptors, that is, NMDA, AMPA, kainate, and metabotropic receptors. Activity-dependent, glutamate-mediated synaptic modification is the main model for long-term plasticity leading to learning and memory in the brain including the hippocampus [11].

The hippocampus plays important roles in long-term memory and spatial navigation. Hippocampal neurons can modify the strength of their connections after brief periods of strong activation. This phenomenon, known as long-term potentiation (LTP) can last for hours or days and has become the best candidate mechanism for learning and memory. 

**Figure 2:**
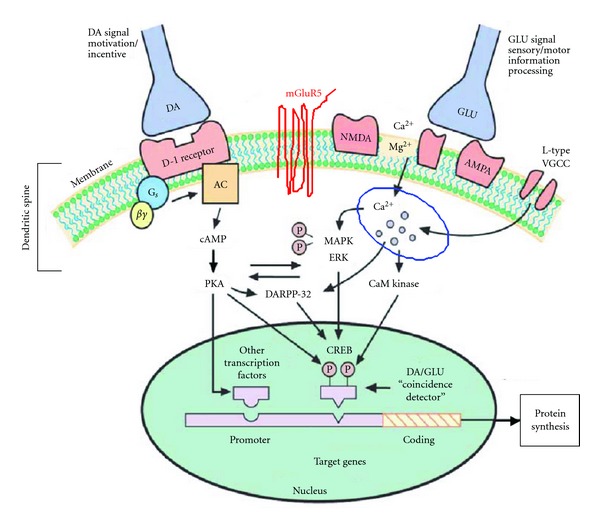
Colocalization of dopamine receptors and glutamate receptors leads to activation of intracellular transduction mechanisms, induction of regulatory transcription factors, and ultimately long-term changes in cellular plasticity in the hippocampus. AC: adenylyl cyclase, AMPA: *α*-amino-3-hydroxy-5-methyl-4 isoxazolepropionic acid receptor, CREB: cAMP-response element binding protein, DA:dopamine, DARPP-32: Dopamine, cAMP-regulated phosphoprotein of 32,000 kDa, ERK: extracellular signal-regulated kinase, GLU: glutamate, GluR5:Metabolic glutamate receptor type 5, MAPK: mitogen-activated protein kinase, NMDA: N-methyl d-aspartate receptor, PKA: protein kinase A, VGCC: voltage-gated calcium channel. (Adopted and modified from [13]).

Additional key elements to the plasticity inherent to the brain reward circuits are dopamine (DA) and their receptors. A critical structural feature pertinent to their contribution to reward learning is the converging projection of both glutamatergic and dopaminergic inputs on the same dendritic spines [12], that is, the colocalization of dopaminergic and glutamatergic terminals in close proximity on the same dendritic spines. Integration of dopaminergic and glutamatergic signals at the cellular and molecular levels is a fundamental process underlying long-term plasticity and reward-related learning. Thus, cells that receive both dopaminergic and glutamatergic signals act as coincidental detectors in associative learning. Hippocampal CA1 pyramidal neurons satisfy this condition as they express colocalization of dopamine receptors and glutamate receptors. In addition, the well-defined anatomy and connectivity of the hippocampus have made it a classical model system to study synaptic transmission and synaptic plasticity (Figure 2).

### 1.4. Endocannabinoid and Ghrelin in Hippocampal Plasticity

The hippocampus is one of a few brain regions that express both cannabinoid receptors (CB1R) and ghrelin receptors in highest concentrations. This suggests that endocannabinoids and ghrelin are prominent neuromodulators for hippocampal neurons. Indeed, endocannabinoids and CB1R have been reported to mediate short- and long-term plasticity in the hippocampus. Endocannabinoids suppress transmitter release either transiently thus causing short-term depression or persistently thus establishing long-term depression. Ghrelin, on the other hand, is reported to enhance long-term potentiation in the hippocampus by increasing the number of spine synapses. This evidence suggests that ghrelin may interact with the glutamate-mediated synaptic transmission and plasticity.

CB1R-dependent short-term depression (STD) can occur on both glutamatergic [14, 15] and GABAergic [16, 17] neurotransmission in all three hippocampal subregions (dentate gyrus, CA3, and CA1). These STDs are caused by the release of endocannabinoids, which is triggered by elevation of intracellular Ca^2+^ concentration in response to the opening of voltage-gated calcium channels [18] and the NMDA-type glutamate receptor [19]. Furthermore, endocannabinoid-driven STD can be enhanced by the activation of the metabotropic glutamate receptors, in particular, by the Group I mGluRs [20].

CB1R-dependent long-term depression (LTD) has been reported in several brain regions including the hippocampus and the brain's reward system. In the striatum, LTD was induced by conjoint activation of group I mGluRs and L-type voltage-gated calcium channels, which induced endocannabinoid release [21]. This study suggested that CB1R activity was necessary for the induction of corticostriatal LTD. The same group further demonstrated that low-frequency presynaptic activity was needed to coincide with activation of CB1R for endocannabinoid-driven LTD [22]. Hippocampal endocannabinoid-mediated LTD was reported of presynaptic origin, and it was blocked by mGluR antagonist, the PLC inhibitor U73122, and the DGL (endocannabinoid synthesizing enzyme) inhibitor RHC-80267 [23]. This LTD was highly localized in a small dendritic area, caused disinhibition, and primed nearby excitatory synapses, thereby facilitating the induction of LTP.

Ghrelin is also reported to facilitate hippocampal LTP. Ghrelin does so by increasing the density of spine synapses in the glutamatergic neurons [4]. However, cellular signaling pathways and molecules involved downstream of the activation of the ghrelin receptor for the generation of new synapses have not been identified. Genes and transcription factors involved in the synapse formation are not identified either. We recently reported an activation of CREB in response to ghrelin application in the hippocampal slice culture and identified the involvement of cAMP-PKA signaling pathways and increased phosphorylation of the NMDA receptor subunit, NR1 [24]. 

Investigation of cellular signal mechanisms for endocannabinoid-mediated synaptic plasticity and ghrelin-mediated metaplasticity has just begun in the hippocampus in order to determine its functional significance in reward-related learning and addiction. One factor that has been most investigated and is involved in the endocannabinoid system, the ghrelin system, and synaptic plasticity is calcium. Ca^2+^ is needed for the production of endocannabinoids, Ca^2+^ is released in response to the activation of the ghrelin receptor, and Ca^2+^ acts as a necessary component in the hippocampal synaptic plasticity mediated by glutamatergic synapses and the subsequent gene expression. Answers to questions such as what signaling pathways communicate or cross-talk each other while the endocannabinoid- and ghrelin systems are activating, and how they might utilize Ca^2+^ as a second messenger, may be a key in understanding the role of the hippocampus in reward-related learning and memory.

## 2. Calcium Ion as an Elementary Signaling Molecule in Synaptic Plasticity

### 2.1. Ca^2+^-Dependent Signaling Pathways Are Involved in Ghrelin- and Endocannabinoid-Mediated Plasticity

In the central nervous system, Ca^2+^ is a ubiquitous and representative second messenger that controls a number of cellular processes including learning and memory. Most forms of synaptic plasticity including LTP have a common requirement for increased intracellular Ca^2+^ initiated postsynaptically by NMDA receptors or presynaptically through voltage-gated Ca^2+^ channels. 

Although the mechanisms by which Ca^2+^ enhances synaptic efficacy have not been fully defined, stimulation of NMDA receptors increases cAMP in the hippocampus [25, 26]. Indeed, several forms of synaptic plasticity including LTP at Schaffer collateral, mossy fiber, and the medial perforant pathways are positively regulated by cAMP. This suggests that Ca^2+^-stimulated adenylyl cyclases may be pivotal for some forms of LTP in the hippocampus [27]. Through the activation of these kinases, Ca^2+^ activates CRE-mediated transcription in neurons by catalyzing its phosphorylation at Ser-133. In addition, Ca^2+^ activates several protein kinases including Ca^2+^/Calmodulin-(CaM-) dependent kinases [28], Erk (extracellular signal-regulated kinase) and MAP (mitogen-activated protein kinase) [29], PKC (protein kinase C) [30], and PKA (protein kinase A) [31]. The sequential activation and translocation of Erk and Rsk2 (p90 ribosomal S6 kinase) by Ca^2+^ were reported to phosphorylate CREB via the activation of PKA. The ghrelin receptor and the cannabinoid receptor are both G-protein coupled receptors. Hence, synaptic plasticity, modulated by these two receptor ligands, that is, ghrelin and endocannabinoids, cannot be discussed completely without the involvement of Ca^2+^ and Ca^2+^-mediated signaling pathways.

### 2.2. Ca^2+^/Calmodulin Protein Kinase

Ca^2+^/Calmodulin protein kinase II-alpha (CaMKII-alpha) has been extensively investigated for the regulation of neuronal excitability. It is well established that CaMKII-alpha translocates to excitatory synapses following strong glutamatergic stimuli that induce NMDA receptor- (NMDAR-) dependent potentiation of many excitatory synapses including long-term potentiation in CA1 hippocampal neurons [32, 33]. Once translocated, CaMKII-alpha leads to the enhancement of surface expression of AMPA receptors (AMPAR) thus maintaining long-lasting LTP via structural changes.

In contrast, in response to moderate NMDAR-activating stimuli, CaMKII-alpha translocates to inhibitory synapses, phosphorylates GABA_A_ receptor (GABA_A_R), and triggers GABA_A_R insertion [34]. Indeed, CaMKII-alpha mediated increase in GABA_A_R-mediated currents has been reported in hippocampal neurons [35, 36]. Translocation of CaMKII-alpha to inhibitory synapses is likely to be an important mechanism for controlling inhibitory synaptic strength. 

Different requirements for translocation to excitatory and inhibitory synapses provide a way for neurons to use this single pathway to potentiate both types of synapses and yet maintain stimulus-dependent specificity in the expression of synaptic plasticity. The differential regulation of CaMKII-alpha targeting to inhibitory and excitatory synapses is dependent on the activation of calcineurin (CaN). CaN prevents CaMKII-alpha targeting to inhibitory synapses when strong stimulation is given to the NMDAR. Similar signaling differences have been associated with the induction of long-term potentiation (LTP) and long-term depression (LTD), which are also attributed to the differential activation of the Ca^2+^-dependent effectors, CaMKII-alpha and CaN [37]. The synapse-specific translocation of CaMKII-alpha provides a mechanism by which activity can couple to the potentiation of inhibitory synapses without producing CaMKII-alpha-dependent LTP at excitatory synapses.

Finally, in addition to directly targeting and regulating the expression of AMPAR and GABA_A_R, Ca^2+^/Calmodulin activates various types of Ca^2+^-dependent kinases and initiates their signaling cascades. CaN, on the other hand, inhibits their signaling cascades. Representative and well-studied kinases are Ca^2+^-dependent adenylyl cyclases.

### 2.3. Ca^2+^-Dependent Activation of cAMP Response-Element Binding (CREB) Proteins

cAMP and CRE (Ca^2+^ response element) are two representative molecules in the CREB/CRE transcriptional pathway. The CRE can integrate Ca^2+^ and cAMP signals. This signaling pathway is implicated in long-term memory (LTM) and transcriptionally-dependent long-lasting LTP (L-LTP) [38] as well as in contextual learning [39]. In addition, CREB is essential for long-term facilitation [40–42]. 

Introduction of targeted genetic manipulations can provide opportunities to dissect memories into its molecular components and identify signal transduction pathways that mediate synaptic plasticity in the hippocampus. The importance of cAMP signal transduction system for learning and memory was demonstrated by the induction of a dominant-negative form of the cAMP response element- (CRE-) binding protein (CREB) that blocked memory formation in Drosophila [41]. On the other hand, induced expression of an activator isoform of dCREB2 enhanced LTM [43]. Recent transgenic mice studies show that a reduction in cAMP-dependent protein kinase (PKA) activity causes defects in L-LTP, spatial memory, and long-term contextual fear conditioning [44]. Increasing evidence suggests that cross-talk between the Ca^2+^, Erk/MAP kinase, and cAMP regulatory pathways may play a pivotal role for some forms of synaptic plasticity and memory formation [45, 46]. 

## 3. Ghrelin-Mediated Signaling 

### 3.1. Ghrelin Stimulates Hippocampal Learning, Reward Behavior, and Consumption of Substances of Abuse

Increasing evidence indicates that the gut peptide ghrelin facilitates learning behavior and memory tasks. A potential functional role for the ghrelin receptor (also known as the growth hormone secretagogue receptor, GHSR1a) in hippocampal memory was first reported by Diano and colleagues [4]. In this study, spine synapses were shown to increase in response to peripheral administration of ghrelin. The functional significance of this anatomical change was explained as that long-term potentiation (LTP) was enhanced and the performance of a hippocampus-dependent maze task was improved. Ghrelin-mediated metaplasticity in the hippocampus may allow animals to adopt food-searching strategies appropriate to their environment by locating, remembering, and recalling whether or not all the available food was consumed [47]. However, the neuroanatomical network integrating ghrelin into memory functions is not well understood.

Intrahippocampal injection of ghrelin was found to improve memory retention when ghrelin was administered before the training session of a step-down behavioral test to wire floor on which a scrambled foot shock was given (inhibitory avoidance). In contrast, when ghrelin was administered during the training session, no improvement was detected on the memory retention [48]. This finding suggested that ghrelin could modulate molecular and/or cellular signaling involved in memory acquisition and/or consolidation, but not in memory retrieval. 

Ghrelin activates the mesolimbic dopamine system and modulates reward and motivational behavior. Direct injection of ghrelin in this system stimulates food intake in a dose-dependent manner [49]. A cellular mechanism for the action of ghrelin was explained as that ghrelin stimulated VTA dopamine neurons via an increase in glutamate release [50]. However, the origin of glutamatergic inputs remains unknown. The signaling pathways through which ghrelin exerted its effect to activate VTA dopaminergic neurons also remain unknown. Nevertheless, it is worth noting that ghrelin enhanced the hedonic and incentive value of food.

Ghrelin also mediates the rewarding properties of alcohol and drugs of abuse including cocaine. Ghrelin injection into the VTA increased voluntary alcohol consumption in a ghrelin receptor (GHSR1a)-dependent manner [51]. Ghrelin sensitizes cocaine- and amphetamine-induced hyperlocomotion and augments cocaine-conditioned place preference [52]. Additional work suggested that ghrelin might be an important cue in triggering the reinstatement of cocaine-seeking behavior as a positive correlation between serum ghrelin levels and cocaine-seeking behavior was observed [53]. 

In summary, ghrelin is one of the most potent orexigens and affects feeding via the central circuitry. Ghrelin is not only involved in hunger-driven (i.e., metabolic demand-dependent) consummatory behavior, but also in a successful search for food with an interplay with the reward system while utilizing the ghrelin-dependent memory retrieval mechanism. 

### 3.2. Ghrelin-Induced Signaling

Ghrelin is a unique acylated 28 amino acid peptide that was first identified in rat stomach extracts as an endogenous ligand for the growth hormone secretagogue receptor (GHSR, or ghrelin receptor). Ghrelin initiates the release of growth hormone through the activation of Gq proteins [54]. In addition, ghrelin increases appetite and initiates feeding behavior [55]. 

The ghrelin receptor is localized in high concentrations in the hypothalamus [56]. However, the hypothalamus is not the only brain region that expresses the ghrelin receptor. The ghrelin receptor is also highly expressed in the cortex [57] and hippocampus [24, 58]. Immunohistochemical analysis showed that the ghrelin receptor had the highest concentration in the somatic region of the pyramidal cell and to a lesser extent in the apical and basal dendritic regions. This observation is in agreement with a previous report on the ghrelin binding assay, which showed that biotinylated ghrelin was scattered in cell bodies of the principal layer of the hippocampal formation [4]. 

Dense localization of the ghrelin receptor in the hippocampus puzzled scientists for the function of the ghrelin receptor in the hippocampus, because the hippocampus is not the brain region that primarily controls feeding behavior or the release of growth hormone. Accumulating evidence, however, indicated that energy homeostasis is important for synaptic plasticity [59, 60]. High-fat and high-glucose diets, which inhibit ghrelin secretion [61], impair hippocampus-dependent synaptic plasticity and spatial memory [62]. On the other hand, exogenous application of ghrelin dose dependently increased memory retention and anxiety-like behavior [63].

In the hippocampus, circulating ghrelin was reported to cross the blood-brain barrier and enhance synapse formation and LTP in CA1 [4]. This evidence suggested that ghrelin could stimulate hippocampus-dependent learning and memory while feeding behavior was actively induced in the hypothalamus. However, little is known about the cellular and molecular mechanisms of ghrelin-mediated enhancement of neuron plasticity in the hippocampus. We know little about the precise subcellular localization of ghrelin receptors in the hippocampus. Interestingly, in contrast to the finding in CA1, ghrelin-induced potentiation in Dentate Gyrus (DG) was not affected by application of D-APV, a blocker of NMDA receptors [64]. In this experiment, it was found that single ghrelin infusion into the hippocampus caused long-lasting potentiation of both the PS (population spike) amplitude and EPSP (excitatory postsynaptic potential) slope. Ghrelin also strengthened HFS (high frequency stimulation)-induced LTP by preventing the LTP decline. There may be a region-specific difference in the role of ghrelin in hippocampal plasticity. 

A well-accepted key molecule in the induction and maintenance of hippocampal LTP is CREB. Indeed, the family of CREB transcription factors has been suggested to be involved in a variety of biological processes, including the development and plasticity of the nervous system [65]. Nevertheless, it is not completely understood whether ghrelin stimulates CREB and activates its signaling in the hippocampus. 

The upregulation of CREB in response to the administration of ghrelin has been reported in colon epithelial cells [66] and hypothalamic neurons [67, 68]. However, kinases involved in the activation of CREB appeared different depending on the brain regions. In the hypothalamus, activation of PKC delta [69] and calcium calmodulin-dependent kinase IV appeared to be necessary and involved [70]. In the hippocampus, activation of cAMP and cAMP-dependent kinase (PKA) is reported to play a critical role in the phosphorylation of CREB in the CA1 pyramidal cell [24]. In the hippocampus, cAMP/PKA signaling has attracted considerable attention in the induction and late (protein synthesis-dependent) phase of the NMDA receptor-dependent LTP. PKA is suggested to play a gating role in the induction of hippocampal plasticity [71]. Thus, ghrelin's stimulatory effect on cAMP and PKA reveals a novel signaling pathway in CA1 pyramidal neuron plasticity. Furthermore, the finding suggests that ghrelin cross-talks with the molecular mechanism of LTP having PKA as a coincidence detector in the induction and maintenance of hippocampal plasticity, causing amplification of NMDA receptor function for increased activation of CREB. Ghrelin may be an endogenous intrinsic stimulus to facilitate the induction of NMDA receptor-dependent hippocampal plasticity.

The ghrelin receptor is primarily coupled to Gq-type G-proteins [54]. This coupling, however, appears to be relatively labile. In the hypothalamus, ghrelin-induced upregulation of cAMP has been reported, indicating that the ghrelin receptor can couple to Gs protein [68]. In the hippocampus, Cuellar and Isokawa [24] demonstrated that ghrelin activated cAMP and protein kinase A (PKA). These findings suggest a novel Gs-coupled signaling pathway of ghrelin in the hippocampus. 

Infusion of ghrelin time dependently increased the phosphorylation of Akt-Ser473, a downstream molecule of phosphoinositide 3-kinase (PI3K) in the dentate gyrus (DG) of the hippocampus [64]. Interestingly, PI3K inhibitors, but not NMDA receptor antagonist, inhibited ghrelin-induced potentiation of hippocampal long-term plasticity in DG. Although ghrelin had no effect on the induction of HFS-induced LTP, it prolonged the expression of HFS-induced LTP through the activation of extracellular signal-regulated kinases (ERK1 and ERK2) [72]. The Morris water maze test showed that ghrelin enhanced spatial memory, and that this was prevented by pretreatment with a PI3K inhibitor. These findings demonstrated the involvement of additional kinases in the ghrelin signaling and ghrelin-mediated metaplasticity in the hippocampal long-term potentiation. Finally, PI3K inhibitors, wortmannin and LY294002, attenuated both the EPSP slope and PS amplitude by abolishing ghrelin-induced potentiation [64]. Finally, in contrast to these findings reported in DG, the involvement of PI3 K has not been confirmed in the CA1 region of the hippocampus in the ghrelin-mediated increase in hippocampal plasticity.

### 3.3. Activation of Ghrelin Receptors and Increase in Cytosolic Ca^2+^

The ghrelin receptor is coupled to Gq-type G-proteins [54]. The alpha subunit of the Gq/11 family stimulates phospholipase C (PLC) beta subfamily including PLC beta1, PLC beta2, and PLC beta3. PLC beta is connected with transmembrane signaling and plays an important role as producers of the second messengers diacylglycerol (DAG) and inositol 1,4,5-trisphosphate (IP_3_). While DAG stays in the membrane serving as a docking site and activator of protein kinase C (PKC), IP_3_ is translocated into the cytoplasm, where it gates IP_3_ receptor Ca^2+^ channels at the membrane of the endoplasmic reticulum (ER). Therefore, stimulation of the ghrelin receptor is expected to increase cytoplasmic Ca^2+^ ([CA^2+^]_i_) concentrations. 

Ghrelin increased [CA^2+^]_i_ in neuropeptide Y-immunoreactive neurons in the arcuate nucleus (ARC), and the maximal effect was obtained by 1 nM of the peptide [73]. The [CA^2+^]_i_ responses to ghrelin were markedly attenuated by inhibitors of protein kinase A (PKA) and a blocker of N-type Ca^2+^ channels. However, inhibitors of protein kinase C and a blocker of L-type Ca^2+^ channels had no effects. Thapsigargin, an inhibitor of the Ca^2+^ pump for the endoplasmic reticulum (ER) and, consequently, Ca^2+^ release from ER had no effect on ghrelin-induced [CA^2+^]_i_ increases. There are two possible explanations for the role of PKA. First, the basal activity of PKA may be required for ghrelin to produce Ca^2+^ signaling. Second, the ghrelin-GHSR1a system may activate the Gs-adenylate cyclase-cAMP-PKA cascade, which in turn leads to the Ca^2+^ influx and [CA^2+^]_i_ increase. However, there is no report to date to demonstrate whether a ghrelin-induced increase of cytosolic Ca^2+^ stimulates calcium-dependent adenylyl cyclases such as AC1 and/or AC8.

The PKA-mediated facilitation of the Ca^2+^ influx and [CA^2+^]_i_ increase has been indicated in the cardiac muscle [74] and in pancreatic *β*-cells [75]. It has been shown that PKA is indispensable for CREB phosphorylation and cAMP response element-mediated gene expression in the hypothalamic neuropeptide Y neurons in the fasted state [76]. Ghrelin could couple fasting to the activation of PKA because the release of this peptide is greatly stimulated by fasting [77]. Estrada and Isokawa [78] showed increased expression of phosphorylated CREB in the fasted rat hippocampus and suggested that an elevated plasma concentration of ghrelin was responsible for the stimulation of CREB activity.

### 3.4. Ghrelin Stimulates CREB Phosphorylation

A study conducted by Cuellar and Isokawa [24] directly tested the prediction proposed by Estrada and Isokawa [78] that fasting elevated hippocampal ghrelin levels and stimulated CREB activity resulting in an increased expression of phosphorylated CREB in the hippocampal neuron. The level of CREB activity was assessed by the immunohistochemical identification of phosphorylated CREB (pCREB) and quantified using an autosegmentation tool provided by imaging software. Ghrelin (200 nM) increased pCREB 4-fold compared to control. The effect of ghrelin was mediated by the ghrelin receptor, as the receptor antagonist L-Dys3-GHSR-6 (100 *μ*M) reduced the expression of pCREB. This finding demonstrated the hippocampal CREB activity was under the regulation of ghrelin. 

The cAMP/protein kinase A (PKA) signaling cascade is necessary for CREB activation and CRE-mediated gene transcription [79]. Therefore, it was examined whether PKA was involved in the ghrelin-induced phosphorylation of CREB in the hippocampus. An inhibitor of PKA (Rp-cAMP, 50 *μ*M) blocked ghrelin's stimulatory effect on the expression of pCREB. This result demonstrated that ghrelin can activate cAMP-dependent kinase, PKA, in hippocampal neurons. 

A primary constituent of the ghrelin receptor is Gq-type G-protein. Gq activation can mobilize cytoplasmic calcium ([CA^2+^]_i_) by translocating IP_3_ to the endoplasmic reticulum and initiate a release of Ca^2+^ from stores. An increase in cytosolic Ca^2+^ can stimulate cAMP production via the activation of Ca^2+^-dependent adenylyl cyclases. Thus, it was tested whether the IP_3_ receptor was involved in the ghrelin-induced increase of pCREB in the hippocampus. Preincubation of the hippocampal slices with 5 *μ*M of Xestospongin-C, a specific antagonist of the IP_3_ receptor, reduced CREB activities. However, it was not selective for inhibiting the ghrelin-induced upregulation of pCREB immunoreactivity [24]. Further studies may be required to address a role of store-released Ca^2+^ in the effect of ghrelin in the hippocampal CREB expression. Combined increases in the second messenger cAMP and calcium have been emphasized as critical in initiating and altering hippocampal gene expression.

PKA has many well-characterized cAMP-dependent roles in cell physiology, which includes the phosphorylation of the NMDA receptor [80]. Phosphorylation potentiates NMDA receptor function and increases receptor-mediated currents [81]. The increased current permits an enhanced Ca^2+^-permeation through the NMDA receptor and facilitates the induction of synaptic plasticity by promoting CREB signaling. Cuellar and Isokawa [24] examined whether ghrelin-mediated CREB expression involved the NMDA receptor.

### 3.5. Ghrelin Enhances NMDA Receptor Phosphorylation

A competitive antagonist of the NMDA receptor, APV, and a specific antagonist of the NMDA receptor subunit NR2B, ifenprodil, both inhibited the ghrelin-mediated increase in pCREB expression [24]. The findings suggest that activation of PKA, NMDA receptor, and NR2B is necessary in ghrelin-mediated stimulation of CREB. It has been well accepted that PKA phosphorylates the NMDA receptor. Phosphorylated NMDA receptor increases channel activities. 

The increased current permits an enhanced Ca^2+^-permeation through the NMDA receptor-channel and facilitates the induction of synaptic plasticity promoting CREB signaling. NR2B is immunologically coexpressed with NR1, suggesting that these two subunits coassemble in the hippocampus [71]. Codistribution of NR1 and NR2B mRNA was also reported [82]. NR1 is the pore-forming obligatory subunit. Channel function of the NMDA receptor is primarily regulated by the phosphorylation of the NR1 subunit at C-terminal serine residues [83]. NR2B affects channel gating to increase NMDA receptor-mediated currents [84]. 

**Figure 3:**
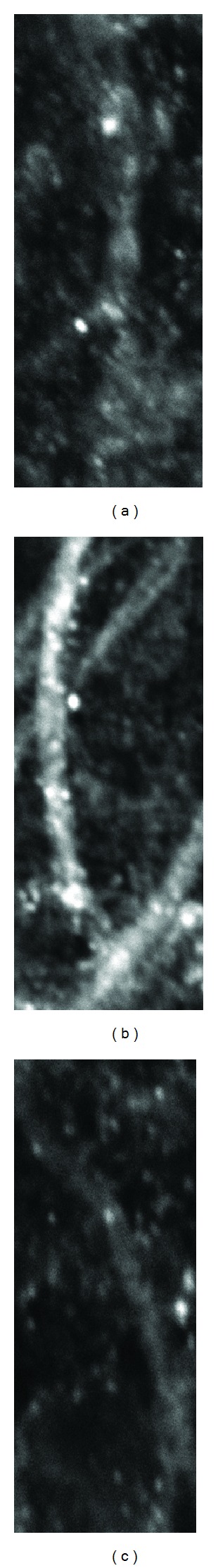
Phosphorylation of NR1 subunit of the NMDA receptor (pNR1) was immunohistochemically detected using an antibody. pNR1 immunoreactivity increased in response to ghrelin (b) when compared with control (a). This effect was blocked by the antagonist of the ghrelin receptor, L-Dys3-GHSR (c). Calibcation: 5 *μ*m. Adopted and modified from Cuellar and Isokawa [24].

Interestingly, NR2B is critically involved in the facilitation of learning consolidation and synaptic plasticity induced by caloric restriction [85]. Caloric restriction can increase plasma ghrelin levels up to 4-fold [86]. These reports further support the interpretation that ghrelin phosphorylates NR2B and, although it may be indirect, enhances the function of NR1. Cuellar and Isokawa [24] examined the magnitude of phosphorylation of NR1 in response to ghrelin. With the use of an antibody against phospho NR1 (pNR1), pNR1 was visualized as small and discrete puncta, primarily on the dendrites of the pyramidal neuron, detected by phallotoxin (Figure 3). The number of immunopositive puncta increased by 46% in response to ghrelin. This finding corroborates a report that the magnitude of NR1 phosphorylation paralleled the magnitude of the NMDA current as well as the magnitude of CREB activation [87]. CaMKII is a likely downstream target of the NMDA receptor in hippocampal synaptic plasticity mediated by the NMDA receptor. Therefore, it is noteworthy that CaMKII-alpha binds to the NR2B subunit of the NMDAR and induces LTP in excitatory synapses [88].

## 4. Endocannabinoid-Mediated Signaling

### 4.1. The Endocannabinoid System in the Hippocampus

The brain cannabinoid system consists of endocannabinoids, the type I and type II cannabinoid receptors (CB1R and CB2R), and a series of intracellular cascades and enzymes that are involved in the synthesis and degradation of endocannabinoids [89]. CB1R is the primary type of the cannabinoid receptor in the brain. CB1R is coupled to Gi/o family of G-protein [90] and is highly expressed in the hypothalamus [91] and the hippocampus [92]. Anandamide and 2-arachidonylglycerol (2-AG) are the main endocannabinoids produced in the central nervous system [93, 94]. Endocannabinoids are synthesized as a result of Ca^2+^-dependent cleavage of phospholipid precursors [89, 95]. Upon synthesis, endocannabinoids are liberated constitutively from plasma membrane without being packaged into vesicles. 

The endocannabinoid system is intimately involved in appetitive and reward-related behavior. In the hypothalamus, the synthesis of endocannabinoids increases during brief starvation and decreases following food intake [96]. Synthesized endocannabinoids stimulate orexigenic neurons, enhance appetite, and facilitate feeding behavior [91]. Evidence suggests that a stomach peptide, ghrelin, may exert its orexigenic effect by stimulating the production of endocannabinoids in the hypothalamus [97]. In this respect, the ghrelin system and the endocannabinoid system work in synchrony in the hypothalamus. Ghrelin levels are high during food deprivation. Expression of CB1R in the hypothalamus also increases during fasting [98]. 

In the hippocampus, endocannabinoids can be produced independently of ghrelin in excitatory and inhibitory neurons as a result of their intrinsic and receptor-mediated activities. Activity-dependent production of endocannabinoids modulates synaptic plasticity by regulating neurotransmitter release [99, 100]. Although there is no report to date that ghrelin induces the synthesis and release of endocannabinoids in the hippocampus, ghrelin crosses the blood-brain barrier and enters into the hippocampus [4]. The rate of ghrelin crossing the blood brain barrier is facilitated by fasting [101]. Indeed, in the amygdala, activation of adenylyl cyclase, cAMP, and PKA induced a release of anandamide [102]. The cAMP/PKA signaling cascade is the pathway that is reported to be activated by ghrelin in the hippocampus [24]. This suggests a likely scenario that ghrelin can stimulate the production of endocannabinoids in the hippocampus. What might be the functional significance of ghrelin-mediated production of endocannabinoids in the hippocampus, considering the fact that the hippocampus does not directly control feeding behavior? If a fasted state is perceived as one of the most stressful conditions, the endocannabinoid system in the hippocampus and amygdala might serve as a stress recovery system not only emotionally but also practically by providing food-acquiring strategies. 

### 4.2. Ca^2+^-Dependent Synthesis of Endocannabinoids in Neurons

Production of endocannabinoids requires participation of cytosolic calcium. However, to date, a specific source of the Ca^2+^ responsible for the synthesis and release of endocannabinoids has not been unequivocally determined. Indeed, a study with photolysis-induced release of caged Ca^2+^ in the cytosol demonstrated that a nonspecific elevation of [CA^2+^]_i_ may be sufficient to synthesize endocannabinoids [103, 104]. 

Ca^2+^-dependent synthesis of endocannabinoids often starts with neuronal depolarization, which activates voltage-gated Ca^2+^ channels (VGCCs) or Ca^2+^-permeable receptor channels. Indeed, voltage-gated calcium channels [18, 105], NMDA receptor channels [19], and calcium-permeable AMPA receptor channels [106] have been proposed as a source of calcium for the synthesis of endocannabinoids. An elevation of cytosolic calcium ([CA^2+^]_i_) caused by the opening of these channels could contribute by themselves to the synthesis and release of endocannabinoids. However, the process can often be amplified by accompanied activation of G-protein-coupled receptors. 

**Figure 4:**
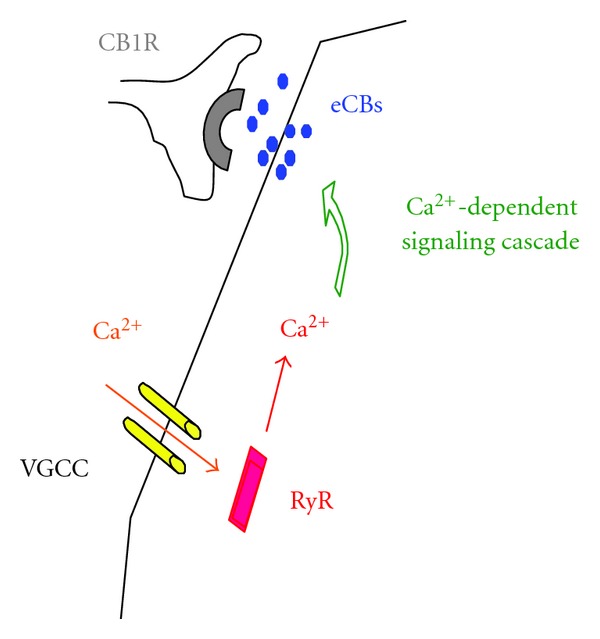
Activation of RyR can provide Ca^2+^ to a distant mobilization site of eCBs away from the Ca^2+^entry site.

There is a presumed distance between the site of Ca^2+^ entry and the site of endocannabinoid synthesis [103, 104, 107]. This leaves a room for the possibility of store-released Ca^2+^ to participate in the synthesis of endocannabinoids (Figure 4). Major calcium release channels are the inositol 1,4,5-trisphosphate receptor (IP_3_R) and the ryanodine receptor (RyR). Ca^2+^ release from IP_3_R depends on the activation of Gq-protein-coupled receptors. However, Ca^2+^-dependent endocannabinoid-synthesis can occur in the presence of the inhibitors of Gq-protein coupled receptors [20]. This evidence suggested that the synthesis of endocannabinoids can be independent of G-proteins when ample Ca^2+^ is available, although endocannabinoid synthesis is facilitated by the activation of Gq-protein-coupled receptors [100]. On the other hand, Berrout and Isokawa [108] demonstrated a tight functional coupling between L-type Ca^2+^ channels and the ryanodine receptor- (RyR-) mediated Ca^2+^ release in the homeostatic regulation of cytosolic Ca^2+^ and stimulus-induced Ca^2+^ signals in the hippocampal neuron. 

### 4.3. The Ryanodine Receptor (RyR) in Endocannabinoid-Mediated Plasticity. 

RyR-mediated calcium release is reported to occur during action-potential generation in the soma and dendrites of hippocampal neurons. It amplifies action potential-driven calcium signals [109–111]. However, Ca^2+^ release from RyR may not be detected easily in the [CA^2+^]_i_-transient evoked by back-propagating action potentials in dendrites [112] or some forms of somatic depolarization [113] due to a net Ca^2+^ uptake by the stores, mainly by the endoplasmic reticulum (ER) [114]. 

The activation of RyRs starts at a [CA^2+^]_i_ between 0.1 and 1 *μ*M and peaks at several *μ*M, although above 10 *μ*M of [CA^2+^]_i_ may inhibit the activation of RyRs [115]. This concentration curve agrees with the reports by Wang and Zucker [104] and Brenowitz and Regehr [116], who demonstrated that a concentration of [CA^2+^]_i_ necessary for the induction of endocannabinoid synthesis might require a *μ*M range. RyR-mediated calcium release is often graded in proportion to the intensity of depolarization and has an apparent threshold for initiation [115, 117]. Similarly, effective induction of calcium-dependent endocannabinoid synthesis requires a threshold level of [CA^2+^]_i_ for initiation [116, 118]. Hence, the functional properties of the RyR-mediated calcium release can readily explain some properties of endocannabinoid synthesis.

Ryanodine receptors (RyRs) are abundantly expressed in the hippocampus. Three isoforms (RyR1, RyR2, and RyR3) are identified in hippocampal neurons [119]. Early in development when neurons undergo dynamic cytodifferentiation and synaptogenesis, RyR3 and RyR1 are highly expressed in the CA1 subfield of the hippocampus. RyR2, on the other hand, increases postnatally and remains high in the adult. The dentate gyrus maintains a high level of RyR1 in the adult. These results suggest that RyRs could contribute to the synthesis of endocannabinoids from the early stage of development throughout the adult. Cultured hippocampal neurons and slices express RyRs similarly in the pattern of neuroanatomical localization to that of in vivo preparations [17, 108, 120, 121]. 

Isokawa and Alger [120] showed that, in CA1 pyramidal cells, a depolarizing voltage step, that can produce endocannabinoid signaling, caused Ca^2+^-induced Ca^2+^ release (CICR) by activating the ryanodine receptor (RyR). When CICR was blocked, the remaining increase in [Ca^2+^]_i_ in response to the same depolarization was less effective in generating endocannabinoid signals. This evidence suggests that voltage-gated Ca^2+^ entry raises local [CA^2+^]_i_ sufficiently to activate local RyRs and that the resulting CICR plays a critical role in initiating endocannabinoid mobilization. 

RyR1 has direct protein-protein interactions with L-type VGCC in neurons [122]. RyR2, on the other hand, forms functional coupling with L-type VGCC. There is no known relationship of RyR3 to VGCCs. However, RyR3-deficient mice show super-enhanced LTP suggesting that RyR3 appeared to attenuate LTP in postsynaptic neurons in the hippocampus [123, 124]. Furthermore, electrophysiological and pharmacological studies have suggested that RyRs are necessary in some forms of glutamatergic [125] and GABAergic LTD [126]. GABAergic LTD is a long-lasting depression of GABAergic inhibition that is mediated by mGluR5 and is well accepted as a cellular model of addiction. Therefore, it may be plausible to hypothesize that mGluR5 might interact with RyRs in the induction of GABAergic LTD, and that RyR-mediated and Ca^2+^-dependent synthesis of endocannabinoid can be modulated by mGluR5. 

### 4.4. Gq Protein-Coupled Receptors and the Ryanodine Receptor

Metabotropic glutamate receptors (mGluRs) can increase intracellular Ca^2+^ concentration via ryanodine-sensitive Ca^2+^ stores in neurons [127]. The mGluR-mediated increase in intracellular Ca^2+^ concentration can activate Ca^2+^-sensitive K^+^ channels and Ca^2+^-dependent nonselective cationic channels. These mGluR-mediated effects often result from mobilization of Ca^2+^ from ryanodine-sensitive, rather than IP_3_-sensitive, Ca^2+^ stores. 

Reports have been accumulating on the group-I mGluRs, primarily coupled to Gq proteins, that activate RyRs independently of IP_3_-mediated Ca^2+^ mobilization. In cerebellar granule cells, the activation of mGluR1 triggers Ca^2+^ entry from L-type Ca^2+^ channels in a ryanodine-dependent manner in the presence of the IP_3_R blocker, heparin and xestospongin C, via a pertussis-toxin-insensitive G protein [128, 129]. One of the mGluR-associated proteins that provides putative molecular substrate for a functional interaction with the ryanodine receptor is the Homer protein family. Homer proteins may physically link mGluR to IP_3_R as well as to RyRs, and this link can be extended to L-type Ca^2+^ channels [130]. This suggests that mGluR (especially mGluR1) might trigger a functional coupling between RyRs and L-type Ca^2+^ channels, which is reminiscent of the cross-talk that exists between RyR1 and L-type Ca^2+^ channels in skeletal muscle cells. Interestingly, muscarinic ACh-receptor stimulation does not mimic the mGluR1-receptor-induced coupling between RyRs and L-type Ca^2+^ channels [128], suggesting that some kind of functional specificity exists in the coupling between these two types of receptors (mGluR and mAChR), intracellular Ca^2+^ stores (IP_3_R and RyRs), and plasma membrane Ca^2+^-permeable channels.

The group-I mGluRs (mGluR1 and mGluR5) display distinct distributions. mGluR1a-receptor immunoreactivity is the strongest in the cerebellar Purkinje cell. On the other hand, in the hippocampus, mGluR1a immunostaining is strong in interneurons of the CA1 region and in some CA3 pyramidal cells, weaker in dentate granule cells, and absent in CA1 pyramidal cells [131, 132]. However, CA1 pyramidal cells show strong mGluR5 immunoreactivity with the highest density on dendritic spines [132]. The distribution of IP_3_R and that of RyRs also show differential localization. In the cerebellar Purkinje cells, prominent levels of IP_3_R exceed the density of RyRs. In the hippocampus, IP_3_Rs are most concentrated in the pyramidal cells of CA1, with substantially fewer in CA3 and dentate. RyRs display an inverse pattern: the highest concentrations are in the dentate gyrus and CA3 region [119]. 

mGluR5 is coupled to Gq/G11 type of G-protein. mGluR5 is localized at excitatory synapses together with ionotropic glutamate receptors such as AMPA receptors and NMDA receptors. mGluR5 can exert long-term modification of AMPA/NMDA receptor-mediated long-term potentiation (LTP), a cellular form of learning. mGluR5 also induces long-term depression (LTD) in both glutamatergic [133] and GABAergic [23, 134] neurons. GABAergic LTD is a long-lasting depression of GABAergic inhibition. When GABAergic LTD occurs, GABA neurons are constantly depressed and thus hypoactive. As a result, the amount of transmitter release (in this case, GABA release) is chronically reduced. This is exactly the same state that is induced by the repeated administration of cannabinoid or cocaine in the drug-sensitive regions of the brain. Most importantly, this form of LTD requires the involvement of RyRs and endocannabinoids in its maintenance. In addition, the requirement of the cAMP and PKA signaling has been indicated [135]. 

Persistent long-term synaptic plasticity requires activation of a new signaling pathway by additional stimuli [136]. Sequential stimulation of separate signaling pathways involving different kinases and G-protein coupled receptors effectively amplifies and maintains longer-lasting plasticity by recruiting a new cascade of proteins. Therefore, it may be plausible to hypothesize that long-lasting changes in hippocampal glutamatergic plasticity may occur during the maintenance phase of GABAergic LTD. Indeed, Chevaleyre and Castillo [137] reported that, when LTD was induced in GABAergic neurons, LTP at excitatory synapses was enhanced. However, this enhanced LTP was conditional to the induction of LTD that was mediated by mGluR5 and CB1R. 

The contribution of a specific mGluR subtype, mGluR5, to the behavioral effect of substances of abuse is striking because it exclusively controls the reinforcing effects of cocaine [138]. Mice lacking the mGluR5 gene do not self-administer cocaine and show no hyperactivity following cocaine treatment despite showing cocaine-induced increases in brain dopamine (DA) levels similar to wild-type (WT) mice. This evidence demonstrates a clear separation of the drug's effect to increase brain DA contents (biochemical effect) from the drug's ability to induce addictive behavior (psychoactive effect). Therefore, mGluR5 appears to be a key molecule that could unleash the drug's psychoactive nature.

Finally, glutamatergic signals are specific to given sensory, motor, or mnemonic information and can be modulated by dopaminergic signals that globally respond to unpredicted, rewarding, or salient events in the environment [139]. For example, in CA1 pyramidal cells in the hippocampus, LTP can be enhanced in the presence of cocaine [140] or endocannabinoids [141]. In these studies, however, the enhancement of LTP was explained as follows: these substances did not directly act on the LTP mechanism to enhance their induction; instead, they downregulated GABAergic inhibition thus causing disinhibition of glutamatergic neurons. Indeed, the downregulation of GABAergic inhibition has long been known to facilitate LTP. Pharmacological blockade of GABA_A_ receptors has widely been used to enhance the induction of LTP. Subsequent studies, however, demonstrated that the way cannabinoids and other substances of abuse down-regulate GABAergic inhibition was not acting on the GABA_A_ receptor; instead, they act on GABAergic terminals and reduce the amount of GABA to be released [100]. Furthermore, Varma et al. [20] reported that the activation of mGluR5 by exogenous application of mGluR5 agonists enhanced the cannabinoid-mediated reduction of GABAergic response in the hippocampal neurons. Although they did not identify the mechanism of this enhancement, Hashimotodani et al. [142] explained the phenomenon as that phospholipase C-beta was activated as a downstream signaling molecule of mGluR5, and phospholipase C beta stimulated an enzyme that initiated the synthesis of endocannabinoids. This study demonstrated that mGluR5 could enhance the amount of endocannabinoid production by acting on the endocannabinoid synthesizing pathways. Gq-proteins are coupled to a variety of metabotropic receptors including the glutamate receptor, acetylcholine receptor, and ghrelin receptor. Future studies are expected to reveal how these receptors may cross-talk each other to facilitate or reduce Gq-protein activated signaling pathways to induce and maintain complexly-interwoven molecular mechanisms for reward-related learning.

### 4.5. Endocannabinoids Negatively Regulate Ca^2+^-Permeable Channel Functions

Endocannabinoids, produced from membrane-bound precursors via calcium and/or G-protein dependent processes, mimic the effects of exogenously applied cannabinoids by activating cannabinoid CB1 and/or CB2 receptors. However, recent studies have indicated that endocannabinoids can produce effects that are independent of cannabinoid receptors. In pharmacologically relevant concentrations, endocannabinoids have been demonstrated to modulate the functional properties of voltage-gated ion channels including Ca^2+^ channels, Na^+^ channels, various types of K^+^ channels, and ligand-gated ion channels such as serotonin (5-HT) and nicotinic acetylcholine (Ach) receptors. Moreover, ion-transporting membrane proteins such as transient potential (TRP) receptor-channels, gap junctions, and neurotransmitter transporters have also been reported to be modulated by endocannabinoids. These modulations are cannabinoid receptor independent. The evidence indicates that, in addition to cannabinoid receptors (CB1R and CB2R), endocannabinoids have separate molecular targets whose activation can alter either the excitability of the neuron or the response of the neuron network. 

2-arachidonoylglycerol (2-AG) and R-methanandamide (nonHydrolyzing form of anandamide) have been reported to inhibit depolarization-induced Ca^2+^ fluxes and specific binding of [^3^H]PN 200-110 (isradipine) to transverse tubule membranes [143]. Anandamide also functionally modulates effects of nifedipine and Bay K 8644 on Ca^2+^ fluxes [144]. On the other hand, synthetic cannabinoids, including CP 55,940, WIN 55,212-2, and Delta9-THC, were ineffective. Experiments using endocannabinoid metabolites suggested that, whereas ethanolamine and glycerol were ineffective, arachidonic acid (AA) inhibited Ca^2+^ fluxes and specific binding of [^3^H]PN 200-110. It appeared that fatty acids containing two or more double bonds were effective in inhibiting depolarization-induced Ca^2+^ fluxes and specific binding of [^3^H]PN 200-110. These results indicate that endocannabinoids directly inhibit the function of voltage-gated calcium channels and modulate the specific binding of calcium channel ligands of the dihydropyridine (DHP) class.

Susceptibility of voltage-gated sodium channels to anandamide and other cannabinomimetic compounds was also investigated. Nicholson et al. [145] reported that anandamide, AM 404, and WIN 55,212-2 inhibited veratridine-dependent depolarization of synaptoneurosomes and a release of l-glutamic acid and GABA. The binding of [^3^H]batrachotoxinin A 20-alpha-benzoate to voltage-gated sodium channels was also inhibited by anandamide, AM 404, and WIN 55,212-2. In addition, anandamide, AM 404, and WIN 55,212-2 markedly blocked TTX-sensitive repetitive firings in cortical neurons. None of the inhibitory effects demonstrated on the sodium channels were attenuated by the CB1 receptor antagonist AM 251. The action of Anandamide was reversible and its effects were enhanced by the inhibitors of fatty acid amidohydrolase. These results suggest that anandamide has a novel signaling pathway for modulating voltage-gated sodium channels independent of the cannabinoid receptor while adding a new mechanism of depressing synaptic transmission in the brain by damping neuronal capacity to support action potentials and reducing evoked release of excitatory and inhibitory transmitters.

Membrane lipids have been demonstrated to be capable of converting A-type K^+^ channels into delayed rectifiers and vice versa. Phosphoinositides remove N-type inactivation from A-type K^+^ channels by immobilizing the inactivation domains. Conversely, arachidonic acid and anandamide endow delayed rectifiers with rapid voltage-dependent inactivation [146]. Similarly, the function of alpha4/beta2 nicotinic acetylcholine receptors (AChR) was reported to be inhibited by anandamide [147]. Anandamide significantly reduced the maximal amplitudes of ACh-induced currents and increased the desensitization of the currents. The effects of anandamide were neither replicated by the exogenous cannabinoid delta9-tetrahydrocannabinol nor reversed by the selective CB1 receptor antagonist 5-(4-chlorophenyl)-1-(2,4-dichlorophenyl)-4-methyl-*N*-(piperidin-1-yl)-1*H*-pyrazole-3-carboxamide (SR-141716A), suggesting that anandamide directly inhibits the function of alpha4/beta2 nAChRs in a CB1 receptor-independent manner.

**Figure 5:**
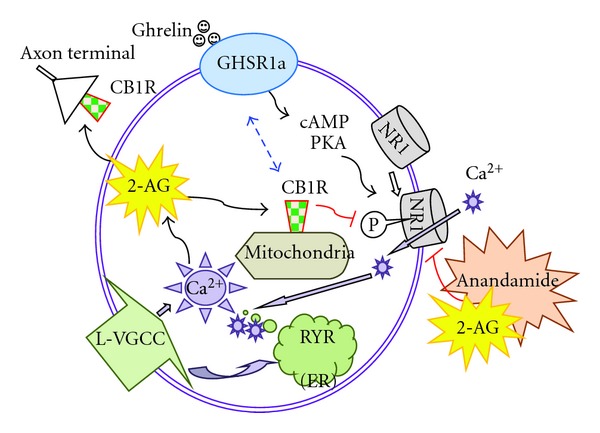
Endocannabinoids negatively regulate ghrelin-induced enhancement of synaptic receptor functions by inhibiting the phosphorylation of NR1. CB1R (type 1 cannabinoid receptor), GHSR1a (type 1a ghrelin receptor, aka growth hormone secretagogue receptor), PKA (protein kinase A), RYR (ryanodine receptor), ER (endoplasmic reticulum), L-VGCC (L-type voltage-gated calcium channel), 2-AG (2-arachidonoyl glycerol).

The NMDA receptor has a well-known Ca^2+^ permeable channel. The activation of the NMDA receptor channel is depolarization dependent. Cuellar and Isokawa [24] reported that endocannabinoids, both 2-AG and anandamide, inhibited the function of the NMDA receptor by inhibiting the phosphorylation of NR1 subunit in the CA1 pyramidal cells of the hippocampus. Interestingly, the inhibitory effects of endocannabinoids were exerted only on the portion of phosphorylation that was enhanced by ghrelin. Indeed, ghrelin had a stimulatory effect on the NR1 phosphorylation. Thus, it can be explained that 2-AG and anandamide exert inhibitory modulation on the phosphorylation of NR1 subunit only in the presence of ghrelin. However, the mechanism of inhibition was different between 2-AG and anandamide. 2-AG exerted its inhibition through the activation of CB1R, while anandamide did so independently of CB1R and the vanilloid receptor (TRPV). This finding identifies the NMDAR as a direct molecular target of endocannabinoids (Figure 5). 

Anandamide and 2-AG are representative endocannabinoids and agonists of the Type 1 CB1R. While endocannabinoids and ghrelin have been shown to synergistically stimulate feeding behavior in the hypothalamus, the contribution of endocannabinoids and CB1R in the hippocampal neuron plasticity has been explained independently of ghrelin. Considering the fact that ghrelin and endocannabinoids are both involved in hippocampal plasticity, future investigation may reveal a deeper understandings of their potential interactions. 

## 5. Genes Involved in Hippocampal Appetitive Learning

There are numerous genes and proteins involved in the induction and maintenance of hippocampal learning. In this section, I will focus narrowly on recently reported new genes that have particular relevance to hippocampal appetitive and motivational learning.

Ghrelin is a stomach-derived peptide that increases food intake through the activation of AMP-activated protein kinase (AMPK) and cAMP response element-binding protein (pCREB). Its regulation by nutritional status has recently become controversial because there are two forms of ghrelin, that is, acyl-ghrelin and des-acyl ghrelin [148]. Studies using new technologies for separately detecting both isoforms indicate that circulating des-acyl ghrelin increases significantly with fasting, whereas acyl-ghrelin levels are not changed over the course of fasting [148, 149]. Most of the effects of ghrelin are exerted through the growth hormone secretagogue receptor 1a (GHSR1a or the ghrelin receptor) [54]. The orexigenic effect of ghrelin is mediated by AMPK, a key upstream master regulator of lipid metabolism [150, 151]. Neuropeptide Y is the best known protein that increases in its expression in response to ghrelin and ghrelin-induced CREB activation in the hypothalamus. However, what proteins may be expressed in the hippocampus, in response to ghrelin and the ghrelin-induced CREB activation, is not well understood. Increases in spine synapses in response to ghrelin may indicate the expression of some types of cytoskeletal proteins. However, the molecular events regulating AMPK phosphorylation after the activation of the ghrelin receptor are unknown either in the hypothalamus or hippocampus.

Sirtuin 1 (SIRT1) is a deacetylase that acts through the tumor suppressor gene p53. SIRT1 is activated in response to calorie restriction in parallel with the increase of ghrelin. The central pretreatment with Ex527, a potent SIRT1 inhibitor, blunted the ghrelin-induced food intake in rats [152]. Mice lacking p53, a target of SIRT1 action, failed to respond to ghrelin in feeding behavior. Ghrelin failed to phosphorylate hypothalamic AMPK when rats were pretreated with Ex527, as it did in p53 KO mice. Interestingly, central administration of AICAR, a potent AMPK activator, increased food intake in p53 KO mice suggesting that the SIRT1/p53 pathway appears to be specifically mediating the orexigenic action of ghrelin, as the blockade of this pathway did not modify ghrelin-induced growth hormone secretion. 

In the hippocampus, Michan et al. [153] showed that SIRT1 was expressed in neurons and necessary and indispensable for cognitive functions including immediate memory, classical conditioning, and spatial learning. They found that the cognitive deficits in SIRT1 knock-out (KO) mice were associated with defects in synaptic plasticity without alterations in basal synaptic transmission or NMDA receptor function. Brains of SIRT1-KO mice exhibited normal morphology and dendritic spine structure but displayed a decrease in dendritic branching, branch length, and complexity of neuronal dendritic arbors. There was also a decrease in extracellular signal-regulated kinase (ERK 1/2) phosphorylation. Furthermore, in SIRT1-KO mice, altered expression was observed in hippocampal genes involved in synaptic function, lipid metabolism, and myelination. In contrast, mice with high levels of SIRT1 expression exhibited regular synaptic plasticity and memory. 

A molecular mechanism for SIRT1 to modulate synaptic plasticity and memory formation was explained by Gao et al. [154] as that it involved a microRNA-mediated mechanism, more specifically, via posttranscriptional regulation of cAMP response-element binding protein (CREB) expression by a brain-specific microRNA, miR-134. SIRT1 normally functions to limit expression of miR-134 via a repressor complex containing the transcription factor YY1. Uncontrolled expression of miR-134 caused by SIRT1 deficiency results in the downregulation of CREB expression and brain-derived neurotrophic factor (BDNF), thereby impairing synaptic plasticity. This finding demonstrates a new role of SIRT1 in hippocampal learning and a previously unknown microRNA-based mechanism by which SIRT1 regulates these processes. Studies of the relationship between SIRT1 and the ghrelin-mediated signaling pathways would advance our understanding of molecular mechanisms specific to appetitive and reward-related learning and memory in the hippocampus.

## 6. Conclusions

Cellular signal mechanisms of appetitive and reward-related learning in the hippocampus are a very important and exciting field of science, as they relate directly to our instinctive behavior for survival. Endocannabinoids and ghrelin are two representative molecules that are intimately involved in reward-related behavior. In this paper, I raised the question of how these two orexigens interact with traditional transmitters and receptors that have long been identified essential in basic hippocampal plasticity. Elucidating their signaling pathways and molecules involved will open up further opportunities not only to understand normal brain functions necessary for healthy growth and survival, but also to value them as a potential therapeutic target for the treatment of central nervous system disorders.
